# 657. Impact of Oral Vancomycin Duration on *Clostridioides difficile* Recurrence in Patients Requiring Concurrent Systemic Antibiotics

**DOI:** 10.1093/ofid/ofad500.720

**Published:** 2023-11-27

**Authors:** Kassandra Marsh, Diana Kwiatkowski, Yanina Dubrovskaya, Alyson Katz, John Papadopoulos, Jonathan So, Vincent J Major, Philip Sommer, Sarah Hochman, Serena Arnouk

**Affiliations:** NYU Langone Health, New york city, New York; NYU Langone Health, New york city, New York; NYU Langone Health, New york city, New York; NYU Langone Health, New york city, New York; NYU Langone Health, New york city, New York; NYULH, New York, New York; NYULH, New York, New York; NYU Langone Health, New york city, New York; NYU Langone Health, New york city, New York; NYU Langone Health, New york city, New York

## Abstract

**Background:**

IDSA guidelines for the treatment (TX) of *Clostridioides difficile* infection (CDI) do not provide specific guidance for total duration of oral vancomycin (VAN) TX in patients requiring prolonged concomitant systemic antibiotics (cABX), as limited evidence exists.

**Methods:**

This was a retrospective study of adult patients with an initial episode of CDI from 2017 to 2022 at NYU Langone Health. Patients were included if they received CDI TX with oral VAN (without fidaxomicin or oral metronidazole) and > 72 hours of cABX (categorized as high, medium or low CDI risk). Prescribing of VAN TX was described as standard duration (SD; ≤ 14 days) vs prolonged duration (PD; > 14 days). The primary outcome was CDI recurrence within 8 weeks of TX completion. Secondary outcomes included risk factors for recurrence, and VAN-resistant *Enterococci* (VRE) isolation and all-cause mortality at 6 months.

**Results:**

After screening 1,350 patients, 218 were included (PD n=140, SD n=78). The median age was 65 years (IQR 54-72), the median Charlson comorbidity index was 4 (2-6), 52% were immunocompromised, 32% required intensive care unit admission, and 66% received high-risk ABX with no differences between groups for any baseline characteristic. Non-severe, severe, and fulminant CDI occurred in 39%, 36%, and 25%, respectively. Patients who received PD had significantly more carbapenem use (37% vs 21%, p=0.011), a longer duration of ABX overlap with VAN TX (11 [7-16] vs 8 [6-11] days, p< 0.001), and more infectious disease consultation (69% vs 54%, p=0.023). After ABX completion, 147 (67%) patients continued VAN TX for a median of 7 (5-10) days, and 30 (14%) patients continued VAN prophylaxis for a median of 6 (5-14) days. Recurrence occurred in 20 (9%) patients, isolation of VRE in 14 (6%), and mortality in 21 (10%), with no statistical differences between SD and PD arms. Stopping VAN prior to completion of ABX was identified as a risk factor for recurrence on univariate analysis (OR 4.25, 95%CI 1.214-14.880, p=0.037).

**Conclusion:**

Our findings suggest that in this high-risk cohort, continuation of VAN after completion of systemic antibiotics may decrease risk of recurrence. Prospective studies are needed to determine optimal VAN TX durations and alternative strategies to reduce CDI recurrence in patients who require cABX.

**Disclosures:**

**All Authors**: No reported disclosures

